# Prescriptions

**Published:** 1897-01

**Authors:** 


JAUNDICE.

℞ Pulv. Rhei ................ ℈ss.

Potass Tart ................ ʒj.

Pulv. Cretæ Aromat ....... ℈j.

Aquæ Menthæ Viridis ..... ℥ij.

M. ft. haustus. octavis horis sumendus.—*Dr. Clark.*

ACUTE BRONCHITIS.

℞. Vini Ipecacuanhæ ........ ʒ ij.
Liq. Potassii Citratis ...... ℥ iv.
Tr. Opii Camphoratæ .....
Syr. Acaciæ ............ aa ℥ j.
M. Sig. A teaspoonful thrice daily in first stage of ordinary acute bronchitis.—*Dr. DaCosta.*

AN EXCELLENT HYDRAGOGUE CATHARTIC

℞. Elaterii .................. gr. 1/12

Ext. Hyoscy ............ gr. iv.

M. Ft. pilula.—*St. Mary's Hospital.*

COMPOUND RHUBARB MIXTURE.

One drachm of this mixture contains:

Ext. Rhei. fl ............ Mj.

Ext. Ipecac, fl ........... M⅕.

Sodii Bicarb ........... gr. ij.

Glycerini ................ ʒ ⅖.

Aq. Menth. Pip .......... ʒ ⅗.

M. Dose, ½ to 1 teaspoonful 2 or 3 times a day for children.—*Dr. Squibb.*

